# Authors' Reply

**DOI:** 10.1371/journal.pmed.0030248

**Published:** 2006-05-30

**Authors:** Daniel Herrera, Alessandro Ieraci

**Affiliations:** **1**Weill Medical College of Cornell UniversityNew York, New YorkUnited States of America

We are grateful to Undurti Das for his suggestions regarding the possible mechanisms of action of nicotinamide in response to our research article [ 1, 2]. He points to the metabolism of essential fatty acids and the inflammatory responses observed after ethanol exposure. We, in fact, consider these important steps in the protection afforded by nicotinamide against ethanol-induced apoptosis, but, additionally, other possible mechanisms may be involved. The effects of nicotinamide appear to be very intricate and in need of more studies. For example, the effects of ethanol on delta-6 desaturase in the brain have not been studied in detail. The delta-6-desaturase increase was correlated with, and augmented, vitamin E levels in the brain [ 3]; however, in our preliminary studies, vitamin E did not ameliorate ethanol-induced apoptosis. All of the mechanisms described below do not exclude the importance of those described by Das.

It has been shown that Bax mediates ethanol-induced apoptotic neuronal death in the developing brain [ 4]. Following an apoptotic stimulus, Bax translocates from the cytoplasm to the mitochondrial membrane, where it increases membrane permeability and promotes the release of cytochrome-c. This increase, in turn, induces activation of caspase-3, leading to cell death. Several mechanisms have been described to promote the translocation of Bax. Activation of the c-Jun N-terminal protein kinase (JNK) plays a critical role in Bax translocation [ 5]. Acute ethanol administration increases the activation of JNK in the postnatal cerebral cortex and striatum [ 6]. It has been shown that nicotinamide prevents the activation of JNK and the N-methyl-N-nitrosourea-induced apoptotic cell death in rat photoreceptor cells [ 7].

Inhibition of the phosphatidylinositol-3-kinase (PI3-K)/ Akt pathway is another cell death mechanism associated with the translocation of Bax [ 8]. Administration of ethanol decreases the activation of Akt in the developing postnatal rat brain [ 6]. Nicotinamide is able to increase the activation of Akt in primary hippocampal neuronal cell cultures, and this is necessary to protect against oxygen-glucose-deprivation (OGD)-induced apoptosis [ 9].

Several studies have pointed out that ethanol exposure increases the generation of reactive oxygen species in the developing brain [ 10]. Nicotinamide has been shown to act directly at the mitochondrial level by preventing the enhancement in mitochondrial permeability transition (MPT) pore opening and the release of cytochrome-c following exposure to OGD [ 9].

Cellular energy metabolism is another significant factor that controls MPT pore formation, as the maintenance of mitochondrial membrane potential is an ATP-facilitated process. During oxidative stress, depletion of NAD, necessary for the production of ATP, is a critical step in precipitating cell death due to compromised energy supply. Nicotinamide administration increases the amount of NAD in the brain, and prevents its depletion and the consequent decrease of ATP [ 11].

We have demonstrated that nicotinamide prevents the ethanol-induced release of cytochrome-c and the following caspase-3 activation in brain development [ 2].

In conclusion, nicotinamide can prevent neuronal cell death by acting at different and probably complementary molecular levels.
